# RETRACTION: Risk Factors for Surgical Site Infection After Percutaneous Endoscopic Lumbar Discectomy

**DOI:** 10.1111/iwj.70609

**Published:** 2025-04-21

## Abstract

**RETRACTION:** XiaoB. , ChenT.‐Y. , ZhaoQ. , ZhaoM. , YangG.‐Q. , ZhongX.‐H. , and XuY.‐Z. , “Risk Factors for Surgical Site Infection After Percutaneous Endoscopic Lumbar Discectomy,” International Wound Journal 21, no. 4 (2024): e14605, 10.1111/iwj.14605.38149500 PMC10961887

The above article, published online on 27 December 2023, in Wiley Online Library (http://onlinelibrary.wiley.com/), has been retracted by agreement between the journal Editor in Chief, Professor Keith Harding; and John Wiley & Sons Ltd. Following an investigation by the publisher, all parties have concluded that this article was accepted solely on the basis of a compromised peer review process. In addition, further investigation by the publisher found that the authors included incomplete ethical approval information for the study. The editors have therefore decided to retract the article. The authors did not respond to our notice regarding the retraction.

**RETRACTION:** B. Xiao , T.‐Y. Chen , Q. Zhao , M. Zhao , G.‐Q. Yang , X.‐H. Zhong , and Y.‐Z. Xu , “Risk Factors for Surgical Site Infection After Percutaneous Endoscopic Lumbar Discectomy,” International Wound Journal 21, no. 4 (2024): e14605, 10.1111/iwj.14605.38149500 PMC10961887

The above article, published online on 27 December 2023, in Wiley Online Library (http://onlinelibrary.wiley.com/), has been retracted by agreement between the journal Editor in Chief, Professor Keith Harding; and John Wiley & Sons Ltd. Following an investigation by the publisher, all parties have concluded that this article was accepted solely on the basis of a compromised peer review process. In addition, further investigation by the publisher found that the authors included incomplete ethical approval information for the study. The editors have therefore decided to retract the article. The authors did not respond to our notice regarding the retraction.

